# Gender Differences in Outcomes after Non-traumatic Intracerebral Hemorrhage

**DOI:** 10.7759/cureus.5818

**Published:** 2019-10-01

**Authors:** Alexandra Craen, Rohan Mangal, Tej G Stead, Latha Ganti

**Affiliations:** 1 Emergency Medicine, University of Central Florida College of Medicine, Orlando, USA; 2 Emergency Medicine, Johns Hopkins University, Baltimore, USA; 3 Emergency Medicine, Brown University, Providence, USA; 4 Emergency Medicine, Envision Physician Services, Orlando, USA

**Keywords:** stroke, intracerebral hemorrhage

## Abstract

Background

Nontraumatic intracranial hemorrhages (ICH) are serious cerebrovascular events with high morbidity and mortality. They occur in about two million people a year worldwide. While ICH continues to be a focus of research in the medical community, there is little data on the differences in outcomes by gender. We aimed to further investigate these differences in our study.

Methods

This analysis involves a de-identified dataset of all adult patients who presented to one of our hospital system's emergency departments with ICHs as one of the top three discharge diagnoses. This study was considered exempt by our medical school's Institutional Review Board (IRB). Our hospital system comprises over 176 hospitals in the United States with over 8.6 million emergency department visits annually. Logistic regression analyses were performed using JMP 14.1. Outcome variables included the length of stay, mortality, and disposition.

Results

The cohort (n = 8069) comprised 68% Caucasians, 17% Blacks, 5% Asians, and 1% Hispanic. Forty-eight percent of patients were females with a median age of 71 years. Fifty-two percent of patients were males with a median age of 65 years. One-fifth of the cohort (20%) died while another fifth (21%) were discharged home. Thirteen percent joined hospice. Women were significantly more likely to die or join hospice (*p* <0.0001, OR 1.304, 95% CI: 1.183-1.440) even after controlling for age. Women also had a significantly shorter length of stay even when controlled for age (*P* = 0.0002, 95% CI: -1.58 to -0.489, *R*^2^ = 1.5%) with a median of four days for men and three days for women.

Conclusion

The median age for women with nontraumatic ICH is older than men, which could explain their increased rates of mortality and discharge to hospice. However, even after controlling for age, women were significantly more likely to die or be discharged to hospice. Conversely, men and younger patients had a longer hospital stay and a higher likelihood of being discharged home.

## Introduction

Intracerebral hemorrhage (ICH) is a life-threatening stroke caused by the rupturing of blood vessels in the brain. The most common causes of ICH are hypertension, arteriovenous malformations, and trauma, representing 10% to 15% of all strokes and over two million people worldwide annually [1-2]. ICH has high morbidity and mortality with 40% of patients dead at 30 days [3]. Patients experiencing ICH may exhibit headaches, focal neurologic deficits, nausea, and vomiting. ICHs are diagnosed via computed tomography (CT) scans, which have demonstrated high sensitivity for acute ICH, showing few false negatives for diagnosis [4]. In a review of 1,421 patients with nontraumatic ICH, the mortality rate was 31.9% [5]. Some studies suggest that Asian males and older individuals are at a statistically greater risk of acquiring ICH [6]. However, while ICH continues to be a focus of research in the medical community, there is limited data on the differences in outcomes by gender. We aimed to further investigate these differences in our study. (Abstract: Alexandra Craen, Latha Ganti, Michelle F. Wallen, and Paul Banerjee. Gender Differences in Outcomes After Intracranial Hemorrhage. SAEM Annual Meeting; May 2019)

## Materials and methods

This is an IRB-exempt observational cohort study of de-identified patient data obtained from January 1, 2015 to December 31, 2016 from our central billing system comprising over 176 hospitals in the United States with over 8.6 million emergency department visits annually. Inclusion criteria were adults aged 18 years and older, who presented to one of our hospital emergency departments with a diagnosis of non-traumatic ICH in the top three diagnosis codes upon hospital discharge or death. Cohort identification was accomplished using ICD 10 (The International Classification of Diseases, Tenth Revision) codes (I61.9 and I62.9). Outcome variables included the length of stay in days, in-hospital mortality, and disposition. The primary outcome was discharge disposition, categorized by location (home, skilled nursing facility, hospice), and stratified by gender. Statistical analysis consisted of summary statistics (distributions), with medians and interquartile ranges reported for non-normally distributed variables. Linear regression analyses were performed to determine factors predictive of outcome. A *p*-value of <0.05 was considered statistically significant. All statistical analyses were performed using JMP Pro 14.1 for Windows.

## Results

The study cohort (n = 8069) comprised 68% Caucasians, 17% Blacks, 5% Asians, and 1% Hispanics. Forty-eight percent of the cohort were females with a median age of 71 years and an IQR of 58-81 years. Fifty-two percent of patients were males with a median age of 65 and IQR 54-76 years. Overall, 20% died within their hospital stay, 21% were discharged home, and 7.5% were discharged to hospice. Table 1 summarizes the demographics of the cohort.

**Table 1:** Cohort demographics IQR, interquartile range

	Women	Men
Median age (IQR)	71 (58-81) years	65 (54-76) years
Age range	18-90 years	18-90 years
White	69%	67%
Black	17%	17%
Hispanic	1%	1%
Asian	4%	5%

Table 2 summarizes the discharge disposition of the cohort. There were 796 women and 788 men who died in the hospital. There were 355 women and 256 men who were discharged to hospice. For the purposes of analysis, we combined the outcomes of death and hospice, for a total of 1151 women and 1044 men who had this outcome. Using the *z*-test for proportions, women were significantly more often to die or go to hospice than men (*P *< 0.0001, two-tailed). In other words, women had a 30% increased odds of death or in hospice (OR 1.304, 95% CI: 1.183-1.440). 

**Table 2:** Discharge disposition of the cohort

	Women		Men	
Discharge disposition	Count	Percentage	Count	Percentage
Acute care hospital	765	1.8%	650	1.7%
Custodial care	17	~0%	15	~0%
Expired	788	1.8%	796	2.1%
Home	980	2.3%	718	1.9%
Hospice	256	6%	355	9.2%
Inpatient rehabilitation facility	667	15.8%	507	13.2%
Left against medical advice	29	0.6%	19	~0%
Long-term care hospital	120	2.8%	98	2.6%
Nursing facility	431	1.0%	550	1.4%
Other	175	4.1%	133	3.4%
Total	4228		3841	

In total, 718 (18.7%) women and 980 (23.2%) men were discharged home. Using the *z*-test for proportions, women were significantly less often to be discharged home (*P *< 0.0001, two-tailed). In other words, women had 24% decreased odds of being discharged home (OR: 0.7619, 95% CI: 0.6839-0.8490).

Age was also a contributing factor to patients ending up dead or in hospice (*P *< 0.0001). For every one-year increase in age, there was a corresponding 2.4% increased odds of death/hospice (OR: 1.024, 95% CI: 1.021-1.028). Both age (*P* < 0.0001) and gender (*P* = 0.0069) retained statistical significance under a multivariate logistic regression model (*R*^2^ = 0.220).

The median length of stay was three days (IQR: 1-8 days, range: 1-270 days) for women and four days (IQR 1-9 days, range 1-226 days) for men. The trend between age and length of stay was negative; younger patients had a higher length of stay, likely because older patients tended to die sooner. Both age (*P *< 0.0001) and gender (*P *= 0.0002) were significant under this regression model (*R*^2^ = 0.0156).

## Discussion

Several studies have been carried out on ICH patient outcomes, but few have studied how gender may specifically affect these outcomes. Previous studies have shown men are either more likely or equally likely to suffer from ICH compared to women [7]. This is consistent with our cohort, wherein 48% were women and 52% were men.

Women in our cohort had a median age of 71 compared to 65 for men in our study. However, even after controlling for age, women had increased odds of death or hospice compared to men in our study. Women also had shorter lengths of stay compared to men after controlling for age, perhaps owing to the increased hospice admission rate. Several other factors may also contribute to the differences in gender outcomes. The low values of the coefficient of determination R^2^ in our regression analyses suggest that the model does not fully explain the outcomes observed. In other words, additional factors beyond age and gender may impart a more significant contribution. However, we did not have data on other possible predictors in this cohort.

The results for mortality by gender after ICH are conflicting in the literature. Some studies reveal that a combination of both age and sex may contribute to the differences in outcomes after ICH [8]. One study showed women in the United States specifically under age 65 had lower mortality than men [9]. By contrast, a Chinese study showed women had higher mortality and early dependence after ICH [10]. In another study on vascular abnormality-related ICH, female sex was an independent risk factor for poor outcomes [11]. These studies showing negative outcomes for women support our findings.

While the strength of our cohort is in its size and geographic representation, the major limitation of our study was the lack of information available on the volume of hemorrhage, presence of intraventricular extension, and baseline comorbidities such as diabetes. These factors may help determine why poor outcomes have been noted for women after ICH. For example, a large public hospital study showed differences by gender in ICH location, risk factors, and demographics [12]. However, in a robust analysis of 245 patients presenting to an emergency department with nontraumatic ICH, a Mayo Clinic study found that female gender was an independent predictor of early mortality, even after adjustment for stroke severity, hemorrhage volume, intraventricular extension of hemorrhage, serum glucose levels, and age [13]. These data suggest that the differences in gender outcome after ICH may be due to non-physiologic or social factors. The impact of “social capital” has been studied by many investigators and is thought to be a significant factor in how people fare [14-15]. Social capital consists of relationships and connections. As women generally outlive men, they have a higher likelihood of living alone in their later years. This may contribute to the disproportionate difference in discharge disposition noted in our study. In other words, women may not have had someone to go home to for care after their ICH as compared to men and hence, they ended up either in hospice or a skilled nursing facility. However, our study was not designed to study this specific aspect.

Studies on gender differences in ischemic stroke may help us support and better understand our findings. A large study using the China National Stroke Registry showed women were more dependent than men after ischemic stroke after one year, which is similar to the earlier Chinese study mentioned on ICH [10,16]. The American Heart Association published a meta-analysis called the INSTRUCT study that showed women had more severe ischemic strokes compared to men. They attributed this mostly to pre-stroke risk factors and recommended improving pre-stroke health in elderly women. They also recommended further study in the biological origins of these gender differences [17]. Similar recommendations can likely be made for ICH.

It appears that a combination of physiological, social, and geographical components may play a role in differences in outcomes by gender, making it difficult to determine a specific target for improving outcomes in such a devastating diagnosis. Continued research is needed on this important topic.

## Conclusions

Older age and female gender present a statistically significant increased risk of death or discharge to hospice after non-traumatic ICH. Conversely, younger age and male gender resulted in longer hospital length of stay and a higher rate of being discharged home.
